# Piezo1 exacerbates inflammation‐induced cartilaginous endplate degeneration by activating mitochondrial fission via the Ca^2+^/CaMKII/Drp1 axis

**DOI:** 10.1111/acel.14440

**Published:** 2024-11-28

**Authors:** Zhidi Lin, Guangyu Xu, Xiao Lu, Hongli Wang, Feizhou Lu, Xinlei Xia, Jian Song, Jianyuan Jiang, Xiaosheng Ma, Fei Zou

**Affiliations:** ^1^ Department of Orthopedics, Huashan Hospital Fudan University Shanghai China

**Keywords:** apoptosis, cartilaginous endplate degeneration, Drp1, mitochondrial fission, Piezo1, senescence

## Abstract

Mitochondrial homeostasis plays a crucial role in degenerative joint diseases, including cartilaginous endplate (CEP) degeneration. To date, research into mitochondrial dynamics in IVDD is at an early stage. Since Piezo1 is a novel Ca^2+^‐permeable channel, we asked whether Piezo1 could modulate mitochondrial fission through Ca^2+^ signalling during CEP degeneration. In vitro and in vivo models of inflammation‐induced CEP degeneration were established with lipopolysaccharide (LPS). We found increased expression of Piezo1 in degenerated CEP tissues and LPS‐treated CEP cells. The Piezo1 activator Yoda1 exacerbated CEP cell senescence and apoptosis by triggering Ca^2+^ influx. Yoda1 also induced mitochondrial fragmentation and dysfunction. In contrast, the Piezo1 inhibitor GsMTx4 exerted cytoprotective effects in LPS‐treated CEP cells. Additionally, the CaMKII inhibitor KN‐93 reversed Yoda1‐induced mitochondrial fission and restored mitochondrial function. Mechanistically, the phosphorylation and mitochondrial translocation of Drp1 were regulated by the Ca^2+^/CaMKII signalling. The Drp1 inhibitor Mdivi‐1 suppressed mitochondrial fission, then reduced mitochondrial dysfunction and CEP cell death. Moreover, knockdown of Piezo1 by siRNA hindered CaMKII and Drp1 activation, facilitating the redistribution of mitochondrial Drp1 to the cytosol in LPS‐treated CEP cells. Piezo1 silencing improved mitochondrial morphology and function, thereby rescuing CEP cell senescence and apoptosis under inflammatory conditions. Finally, subendplate injection of GsMTx4 or AAV‐shPiezo1 alleviated CEP degeneration in a rat model. Thus, Piezo1 may exacerbate inflammation‐induced CEP degeneration by triggering mitochondrial fission and dysfunction via the Ca^2+^/CaMKII/Drp1 axis.

## Graphical abstract

Schematic illustration of the role of Piezo1 in inflammation‐induced CEP degeneration. Piezo1 promotes Drp1 S616 phosphorylation and mitochondrial translocation, then triggers mitochondrial fragmentation and dysfunction via the Ca2+/CaMKII signalling.

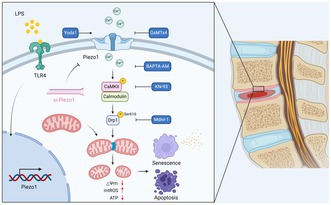

**Abbreviations**
AAV: adeno‐associated virus
ACDF: anterior cervical discectomy and fusion
AF: annulus fibrosus
ATP: adenosine triphosphate
CEP: cartilaginous endplate
ECM: extracellular matrix
IMM: inner mitochondrial membrane
IOD: integrated optical density
IVDD: intervertebral disc degeneration
LPS: lipopolysaccharide
MC: modic change
MMP: mitochondrial membrane potential
MRI: magnetic resonance imaging
mtROS: mitochondrial reactive oxygen species
NP: nucleus pulposus
OMM: outer mitochondrial membrane
ROS: reactive oxygen species
SD: standard deviation

## 1 INTRODUCTION

Cartilaginous endplate (CEP) degeneration is an important initiator of intervertebral disc degeneration (IVDD). IVDD is the primary cause of a series of degenerative spine diseases (Huang et al., 2023). The IVD, which consists of the nucleus pulposus (NP), annulus fibrosus (AF) and CEP, is a fibrocartilaginous tissue connecting two adjacent vertebral bodies. It provides mechanical stability to the spine and enables segmental mobility. The CEP is a layer of hyaline cartilage between the NP and bony endplate. The IVD largely relies on the permeation of the CEP to provide oxygen, glucose and other nutrients through diffusion, as it is considered as the largest avascular organ in the human body. Therefore, the CEP serves as an important nutrient channel for the IVD (Urban et al., 2004). Many pathological stimuli, including inflammation, oxidative stress, mechanical loading and inadequate nutrient supply, can lead to IVDD, which is characterized by excessive disc cell apoptosis, senescence, inflammatory responses and extracellular matrix (ECM) catabolism (Kamali et al., 2021). Chondrocyte senescence and apoptosis are two major characteristics of CEP degeneration. They are barriers to nutrient transport and contribute to the development of IVDD (Xiang et al., 2022; Zehra et al., 2022). Clinical and experimental evidence suggests that infection and inflammation induce CEP degeneration (Dudli et al., 2016). However, the mechanisms underlying inflammation‐induced CEP degeneration remain unclear.

Piezo1 is a mechanosensitive cation channel that facilitates the translation of extracellular mechanical stimulation into intracellular biological signalling cascades, which is termed mechanotransduction (Coste et al., 2010). Piezo1 allows Ca^2+^ influx; thus, Piezo1 can regulate intracellular Ca^2+^ concentrations and mediate downstream Ca^2+^ signalling pathways (Jiang et al., 2021). Accumulating evidence has suggested that Piezo1 is essential for the regulation of osteoarthritis (Lee et al., 2014), IVDD (Wang et al., 2021), osteoporosis (Sun et al., 2019) and fracture healing (Liu et al., 2022). Interestingly, some researchers have reported that the expression level of Piezo1 could be increased by proinflammatory factors, such as bacterial infection, lipopolysaccharide (LPS) and interleukin‐1α (IL‐1α) (Lee et al., 2021; Velasco‐Estevez et al., 2018; Velasco‐Estevez, Rolle, et al., 2020), while most studies have focused on the mechanical activation of Piezo1. Inflammation may affect the gene regulation and function of Piezo1, in turn making cells hypersensitive to mechanical loading and facilitating mechanotransduction (Geng et al., 2021; Lee et al., 2021). The CEP in the spine is constantly exposed to mechanical forces. Aberrant loading and inflammation are crucial risk factors for CEP degeneration and IVDD. Consequently, we hypothesize that Piezo1 may act as a bridge between these two risk factors in the pathogenesis of CEP degeneration.

Mitochondria are vital organelles with double‐layered membranes composed of the outer mitochondrial membrane (OMM) and inner mitochondrial membrane (IMM). Mitochondria are the “power houses” of cells and participate in the regulation of adenosine triphosphate (ATP) synthesis, calcium signalling, reactive oxygen species (ROS) production and cell death (Fontana & Limonta, 2021; McBride et al., 2006). Moreover, emerging evidence has indicated that mitochondrial dysfunction is closely related to chondrocyte senescence (Coryell et al., 2021) and apoptosis (Blanco et al., 2011). As highly dynamic organelles, mitochondria rapidly and continuously undergo fusion and fission, which is termed mitochondrial dynamics. Mitochondrial fusion rescues mitochondrial dysfunction by mixing the contents of partially damaged mitochondria with healthy mitochondria as a form of complementation. In contrast, fission generates small and fragmented mitochondria to facilitate the elimination of damaged mitochondria and the regulation of mitochondrial transport and distribution (Sharma et al., 2019). Although mitochondrial fission is indispensable for cellular homeostasis, it is often aberrantly activated under pathological conditions. Hyperactive fission contributes to mitochondrial fragmentation and dysfunction, ultimately triggering cell death (Quiles & Gustafsson Å, 2022). Previously, we performed a literature review and summarized the role of mitochondrial fission in the pathogenesis of IVDD (Lin et al., 2023). To date, research into mitochondrial dynamics in IVDD is at an early stage. Thus, we examined whether mitochondrial fission is involved in CEP degeneration and explored the underlying molecular mechanisms.

## 2 MATERIALS AND METHODS

### 2.1 Ethics statement

The acquisition of human CEP tissues was approved by the Huashan Hospital Ethics Committee, Fudan University (No. KY2022‐524). All animal procedures were approved by the Animal Care and Use Committee of Fudan University (No. 202208006S).

### 2.2 Human CEP tissue collection, rat CEP cell culture, and treatment

Healthy control CEP tissues were obtained from three patients with Hirayama disease who underwent anterior cervical discectomy and fusion (ACDF). Degenerated CEP tissues were collected from three patients who underwent ACDF because of cervical spondylosis [Table S1]. The degree of IVDD was assessed according to the Pfirrmann magnetic resonance imaging (MRI) grading system, and samples with Pfirrmann grades I and II were regarded as nondegenerated (Pfirrmann et al., 2001).

Isolation of primary CEP cells was performed according to the protocol reported by Piprode et al. (2020). Twelve 8‐week‐old male Sprague–Dawley rats were used. After sacrifice, the thoracic and lumbar vertebrae (T1–T13 and L1–L6) of rats were surgically removed from the back. CEP tissues were excised from the thoracic and lumbar discs, fragmented and digested with 0.2 mg/mL type II collagenase (17,101,015, Thermo Fisher Scientific) for 12 h. The released cells were collected by centrifugation for 5 min at 500*g*. CEP cells were then resuspended in Dulbecco's modifed Eagle's medium (DMEM; PM150210, Procell, China) with 10% foetal bovine serum (FBS; 164,210, Procell, China) and maintained in a humidified incubator at 37°C with 5% CO_2_. The culture medium was changed every 3 days. Second‐ and third‐passage CEP cells were used in subsequent experiments.

To mimic the inflammatory microenvironment of CEP degeneration, 10 μg/mL LPS (Biosharp, China) was used to treat CEP cells for 12 h. Then, CEP cells were treated with 5 μM Yoda1 (MedChemExpress, USA) or 10 μM GsMTx4 (MedChemExpress, USA) for 12 h to investigate the function of Piezo1 in inflammation‐induced CEP cells. To further study the downstream signal transduction of Piezo1, CEP cells were treated with 5 μM Yoda1 plus 10 μM BAPTA‐AM (MedChemExpress, USA), 10 μM KN‐93 (MedChemExpress, USA) or 10 μM Mdivi‐1 (Selleck Chemicals, USA) for 12 h.

### 2.3 RNA interference

The rat Piezo1‐siRNA and its negative control were constructed by HYcell Biotechnology (Wuhan, China). The sequences used were as follows: Rat si‐Piezo1: 5′‐GAUGCUGUAUCAGCUGAAG‐3; Rat si‐NC: 5′‐GAUAUCAUGGCGCUGUAAG‐3′. The CEP cells were transfected with siRNAs using Lipofectamine 3000 (Invitrogen, USA) according to the manufacturer's instructions.

### 2.4 Western blot analysis

Tissue or cells were lysed on ice in buffer containing protease inhibitors (AS1008, Aspen). The protein fractions were collected and separated by SDS–PAGE. Then, the proteins were transferred onto a PVDF membrane (IPVH00010, Millipore). The membrane was blocked with 5% skimmed milk. Afterwards, primary antibodies were added, incubated with the membrane overnight at 4°C and then detected using horseradish peroxidase‐conjugated secondary antibodies. The antibodies used in this study included anti‐Piezo1 (1:500, 15939‐1‐AP, Proteintech Group), anti‐GAPDH (1:10,000, ab181602, Abcam), anti‐cleaved caspase‐3 (1:1000, AF7022, Affinity Biosciences), anti‐Bax (1:2000, #2772, CST), anti‐Bcl‐2 (1:1000, ab196495, Abcam), anti‐RUNX2 (1:1000, 20700‐1‐AP, Proteintech Group), anti‐CaMKII (1:1000, ab134041, Abcam), anti‐p‐CaMKII (Thr286) (1:500, ab171095, Abcam), anti‐Drp1 (1:1000, #8570, CST), anti‐p‐Drp1 (Ser616) (1:500, PA5‐121305, Thermo Fisher Scientific), anti‐Cytochrome c (1:1000, #11940, CST), anti‐β‐Actin (1:10,000, TDY051, Beijing TDY Biotech), and anti‐VDAC1 (1:3000, ab15895, Abcam).

### 2.5 Quantitative real‐time PCR (qRT–PCR)

Total RNA was extracted from cells using TRIpure reagent (EP013, ELK Biotechnology) according to the manufacturer's instructions. The following primers were used: rat GAPDH primers (F: 5′‐AACAGCAACTCCCATTCTTCC‐3′, R: 5′‐TGGTCCAGGGTTTCTTACTCC‐3′) and rat Piezo1 primers (F: 5′‐TGGGGCTCTACGTCTCTATCG‐3′, R: 5′‐GTCTCCCGTACCAAGAAGATG‐3′). GAPDH was used for normalization.

### 2.6 Immunofluorescence (IF) staining

Following the treatments, CEP cells were fixed with 4% paraformaldehyde for 20 min and then permeabilized with 0.5% Triton X‐100 for 20 min. After the cells were blocked with 5% bovine serum albumin, they were incubated with primary antibodies against Piezo1 (1:200, PA5‐106296, Thermo Fisher Scientific) or p‐Drp1 (1:200, PA5‐106169, Thermo Fisher Scientific) at 4°C overnight. After being washed with PBS three times, the samples were incubated with the corresponding secondary antibodies (1:100, SA00013‐2, Proteintech Group) at 37°C for 40 min. The nuclei were stained with 4′,6‐diamidino‐2‐phenylindole (DAPI) (D8417‐1MG, Sigma–Aldrich) for 20 min. Finally, images were observed using a fluorescence microscope (IX51, Olympus, Japan) and fluorescence confocal microscope (LSM880, ZEISS, Germany).

### 2.7 Detection of calcium levels

Intracellular Ca^2+^ levels were detected using the specific Ca^2+^‐sensitive fluorescent indicator Fluo‐4 AM (F14201, Thermo Fisher Scientific) according to the manufacturer's instructions. Briefly, after being treated, CEP cells were incubated with 5 μM Fluo‐4 AM for 30 min at 37°C in the dark. Images were acquired using a fluorescence confocal microscope (LSM880, ZEISS, Germany).

### 2.8 Senescence‐associated β‐galactosidase staining

Senescence‐associated β‐galactosidase staining was performed by using a Senescence Assay Kit (ab228562, Abcam). CEP cells were incubated with senescence working solution according to the manufacturer's protocol. Cellular senescence was determined by monitoring the fluorescence signals of β‐galactosidase using flow cytometry.

### 2.9 Apoptosis assay

Flow cytometry with Annexin V‐FITC/PI was performed to examine CEP cell apoptosis. CEP cells were stained using an Apoptosis Analysis Kit (AO2001‐02P‐H, SUNGENE BIOTECH) according to the manufacturer's instructions. Briefly, after being resuspended, CEP cells were incubated with 5 μL of Annexin V‐ FITC for 10 min in the dark. The CEP cells were then incubated with 5 μL of PI for 5 min in the dark. Finally, the CEP cells were analysed by flow cytometry (BD FACSCalibur, Becton, Dickinson and Company).

Terminal deoxynucleotidyl transferase biotin‐dUTP nick end labelling (TUNEL) staining was used to detect the level of DNA damage in CEP cells. CEP cells were fixed and stained with an In Situ Cell Death Detection Kit (11684817910, Roche) according to the manufacturer's instructions, and the nuclei were stained with DAPI.

### 2.10 Mitochondrial membrane potential (MMP) assessment

MMP was measured by the JC‐1 Assay Kit (C2006, Beyotime) according to the manufacturer's instructions. CEP cells were incubated with JC‐1 working solution for 20 min at 37°C and then washed twice with JC‐1 buffer solution. The stained cells were detected using flow cytometry. The results were analysed by determining the ratio of JC‐1 aggregates to monomers.

### 2.11 Measurement of mitochondrial reactive oxygen species (mtROS) and cellular ROS

Cellular ROS and mtROS were detected using MitoSOX Red (M36008, Thermo Fisher Scientific) and a ROS Assay Kit (S0033, Beyotime), respectively. CEP cells were incubated with MitoSOX Red for 10 min or DCFH‐DA for 20 min at 37°C in the dark. The cells were then washed three times with PBS. Images were obtained using a fluorescence microscope (IX51, Olympus, Japan) and fluorescence confocal microscope (LSM880, ZEISS, Germany).

### 2.12 MitoTracker staining

MitoTracker Red CMXRos (C1049B, Beyotime) was used to stain mitochondria. After treatment, the CEP cell medium was discarded. The cells were incubated with MitoTracker Red solution at 37°C for 20 min in the dark. Images of mitochondrial morphology were captured using a fluorescence confocal microscope (LSM880, ZEISS, Germany).

### 2.13 Measurement of cellular ATP

ATP levels in CEP cells were assessed using an Enhanced ATP assay kit (S0027, Beyotime). CEP cells were lysed with ATP lysis buffer. Then, the lysate was centrifuged at 12,000 × g for 5 min, and the supernatant was reacted with the ATP detection working solution. Luminescence activity was measured by a spectrophotometer (UV‐1600, MAPADA, China), and the ATP level was normalized to the cellular protein concentration.

### 2.14 Establishment of a rat model of inflammation‐induced CEP degeneration

A rat CEP degeneration model was established according to the modified protocols of Rajan et al. (2013) and Yuan et al. (2015). Eight‐week‐old male Sprague–Dawley rats were included in this study. Adeno‐associated virus (AAV)‐mediated knockdown of Piezo1 was used to assess the therapeutic effects of inhibiting Piezo1. The AAVs were constructed by HYcell Biotechnology (Wuhan, China). A total of 24 rats were randomly and equally assigned to four groups: the control group, the LPS group, the LPS + GsMTx4 group and the LPS + AAV‐shPiezo1 group. The rats were anaesthetized by intraperitoneal injection of 40 mg/kg pentobarbital. Except for the control group, they were subjected to coccygeal vertebral drilling through the cortical bone and into the bone marrow percutaneously. Co7/8 was located by digital palpation and selected to construct the CEP degeneration model. Bone drilling was manually performed on the 8th coccygeal vertebral body in the proximity of the Co7/8 disc (subendplate) by using a 22G lumbar puncture needle. Then, a tunnel was created by drilling, and subendplate injection of LPS (10 μg/mL, 25 μL) into the vertebral bone marrow was accomplished by using a microlitre syringe. To treat CEP degeneration, the rats in the LPS group were injected with PBS (25 μL); The rats in the LPS + GsMTx4 group were injected with GsMTx4 (10 μM, 25 μL). The rats in the LPS + AAV‐shPiezo1 group were injected with AAV‐shPiezo1 (1 × 10^10^ v.g/mL, 25 μL). Finally, the animals were returned to their cages with unrestricted activity. The injections of PBS, GsMTx4 or AAV‐shPiezo1 were conducted each week for 1 month.

### 2.15 MRI

The coccygeal vertebrae of rats were examined by MRI (BioSpec 70/30 USR, Bruker, Germany) to assess the signal and structural changes in the sagittal T2‐weighted image. The degree of IVDD was evaluated using the Pfirrmann grading system (Pfirrmann et al., 2001).

### 2.16 Histological staining and immunohistochemistry

The rat samples were collected, fixed, decalcified, dehydrated, embedded in paraffin and cut into 4 μm sections. H&E staining, Safranin O‐Fast Green staining and Alcian Blue staining were conducted to assess the histological degree of degeneration in the discs (Mao et al., 2011). Immunohistochemistry was performed to analyse the expression levels of cleaved caspase‐3. The integrated optical density (IOD) of the immunohistochemical images was quantified using ImageJ software.

### 2.17 Statistical analysis

The results are presented as the mean ± standard deviation (SD). *n* = 3 biological replicates were performed for in vitro experiments and *n* = 6 biological replicates were performed for in vivo experiments. Statistical analyses were performed using SPSS 21.0 and GraphPad Prism 9.0 software. The data were analysed using Student's *t* test for comparisons between two groups and one‐way analysis of variance followed by Tukey's test for comparisons among multiple groups when appropriate. A value of *p* < 0.05 was considered to indicate statistical significance.

## 3 RESULTS

### 3.1 Elevated Piezo1 expression in human degenerated CEP tissues and LPS‐treated CEP cells

**FIGURE 1:**
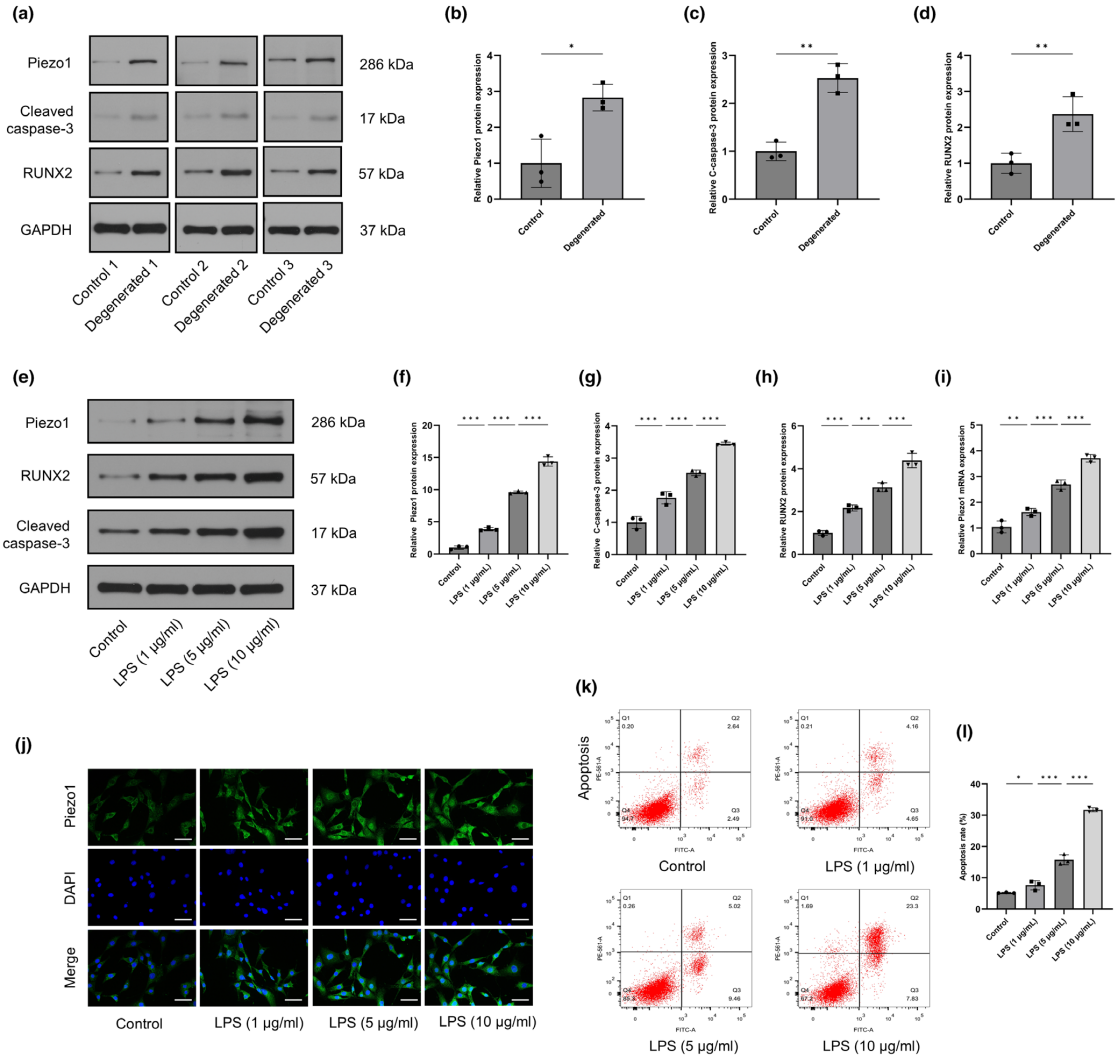
Piezo1 expression was elevated in human degenerated cartilaginous endplate (CEP) tissues and LPS‐treated CEP cells. (a–d) western blot analysis showing the protein expression levels of Piezo1, cleaved caspase‐3 and RUNX2 in human CEP tissues (three control and three degenerated samples). (e–h) The protein expression of Piezo1, cleaved caspase‐3 and RUNX2 in CEP cells was upregulated by LPS. (i) Piezo1 mRNA levels in LPS‐treated CEP cells were measured by qRT–PCR. (j) Immunofluorescence staining of Piezo1 in CEP cells after LPS treatment. Scale bar, 50 μm. (k, l) The effects of LPS on CEP cell apoptosis were analysed by flow cytometry using Annexin V‐FITC/PI staining. (*n* = 3 biological replicates, *p < 0.05; **p < 0.01; ***p < 0.001).

To explore the role of Piezo1 in CEP degeneration, we collected three samples of degenerated CEP tissues from patients with cervical spondylosis and three samples from normal CEP tissues from patients with Hirayama disease. The western blot results confirmed increased expression of apoptosis‐related proteins (cleaved caspase‐3) and calcification‐related proteins (RUNX2) in the degeneration group. In addition, the expression level of Piezo1 was increased in degenerated CEP tissues (Figure 1a–d). Subsequently, LPS, which is widely used in preclinical models of inflammation, was used to mimic the inflammatory microenvironment of CEP degeneration (Rajan et al., 2013). Interestingly, the mRNA and protein levels of Piezo1 in CEP cells were increased by LPS in a dose‐dependent manner, as evaluated by qRT–PCR, western blotting and immunofluorescence staining (Figure 1e,f,i,j). As expected, LPS stimulation resulted in a dose‐dependent increase in the apoptosis rate of CEP cells, which was measured by flow cytometry (Figure 1k,l). Consistently, the expression of cleaved caspase‐3 and RUNX2 was upregulated in LPS‐treated CEP cells (Figure 1e,g,h). Taken together, these data suggested that Piezo1 was correlated with the development of CEP degeneration.

### 3.2 Piezo1 increased inflammation‐induced senescence and apoptosis in CEP cells

**FIGURE 2:**
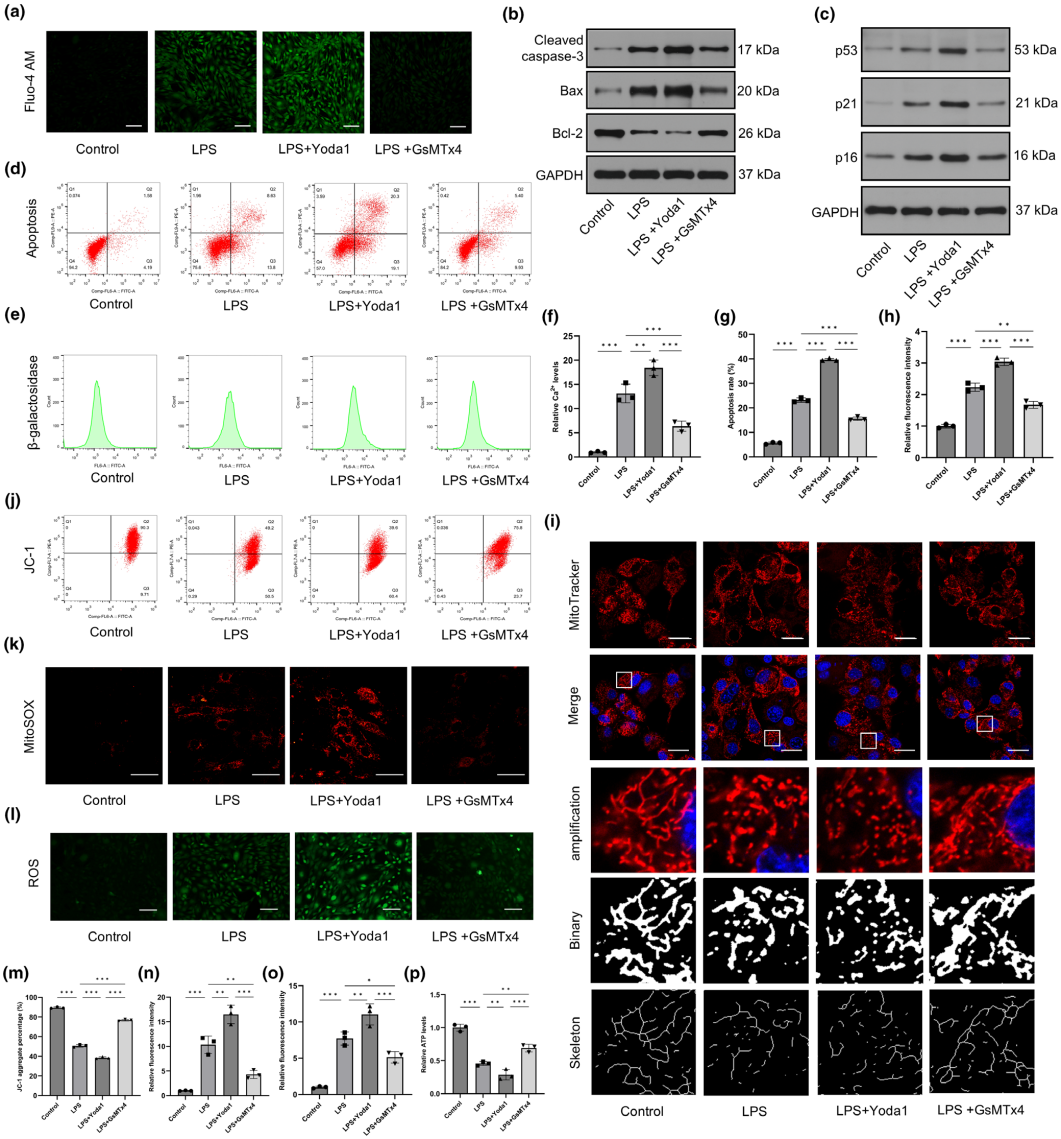
Piezo1 promoted cartilaginous endplate (CEP) cell senescence and apoptosis by triggering mitochondrial fragmentation and dysfunction. (a, f) The influences of Yoda1 and GsMTX4 on intracellular Ca^2+^ levels in LPS‐treated CEP cells were using the specific Ca^2+^‐sensitive fluorescent indicator Fluo‐4 AM. Scale bar, 100 μm. (b) The expression of apoptosis‐related proteins (cleaved caspase‐3, Bax, Bcl‐2) was measured by western blotting. (c) The expression of senescence‐related proteins (p53, p21 and p16) was measured by western blotting. (d, g) The effects of Yoda1 and GsMTX4 on LPS‐induced apoptosis of CEP cells were analysed by flow cytometry with Annexin V‐FITC/PI. (e, h) Flow cytometry with β‐galactosidase staining was used to evaluate the level of CEP cell senescence. (i) The mitochondrial morphology in CEP cells was detected by MitoTracker Red staining. Scale bar, 25 μm. (j, m) The MMP in CEP cells was assessed by flow cytometry using JC‐1 staining. (k, l, n and o) The production of mitochondrial and cellular reactive oxygen species (ROS) in CEP cells was measured by MitoSOX Red and DCFH‐DA staining, respectively. Scale bars, 50 μm (k) and 100 μm (l). (p) Relative ATP contents in LPS‐induced CEP cells after treated with Yoda1 or GsMTx4. (*n* = 3 biological replicates, *p < 0.05; **p < 0.01; ***p < 0.001).

To investigate the function of Piezo1 in CEP cells under inflammatory conditions, the Piezo1 agonist Yoda1 and the mechanosensitive cation channel inhibitor GsMTx4 were used (Velasco‐Estevez, Gadalla, et al., 2020). Piezo1 is a nonselective cation channel that allows Ca^2+^ influx and initiation of the downstream Ca^2+^ signalling pathway. Thus, we assessed whether Piezo1 mediates Ca^2+^ influx in CEP cells by using the specific Ca^2+^‐sensitive fluorescent indicator Fluo‐4 AM. Treatment with Yoda1 increased intracellular Ca^2+^ levels, while GsMTx4 decreased intracellular Ca^2+^ levels in LPS‐treated CEP cells (Figure 2a,f). Furthermore, Yoda1 increased the percentage of apoptotic cells (Figure 2d,g) and the expression level of proapoptotic proteins (cleaved caspase‐3 and Bax) but reduced the expression of the antiapoptotic protein Bcl‐2. In contrast, GsMTx4 suppressed inflammation‐induced CEP cell apoptosis (Figure 2b). Western blot analysis of senescence‐related proteins (p53, p21 and p16) and β‐galactosidase staining indicated that Yoda1 promoted CEP cell senescence, while GsMTx4 exerted the opposite effects (Figure 2c,e,h). Collectively, the results showed that Piezo1 could trigger Ca^2+^ influx and modulate CEP cell senescence and death under inflammatory conditions.

### 3.3 Piezo1 regulated mitochondrial morphology and function in CEP cells

We sought to determine how intracellular calcium concentrations influenced the phenotype of CEP cells. Mitochondria are closely related to Ca^2+^ signalling and cell death. Therefore, we hypothesized that excessive Ca^2+^ influx exacerbates apoptosis by inducing mitochondrial dysfunction. Interestingly, flow cytometric analysis of JC‐1 staining demonstrated that Yoda1 exacerbated the loss of mitochondrial membrane potential (MMP), while GsMTx4 exerted a protective effect on mitochondria in CEP cells after LPS stimulation (Figure 2j,m). Moreover, Yoda1 promoted the production of mitochondrial and cellular ROS, which were detected by using MitoSOX Red (Figure 2k,n) and DCFH‐DA staining (Figure 2l,o), respectively. In contrast, GsMTx4 alleviated mitochondrial and cellular oxidative stress in LPS‐treated CEP cells. The production of ATP was reduced by Yoda1 treatment while GsMTx4 could maintained ATP contents in LPS‐stimulated CEP cells (Figure 2p). Since mitochondria are highly dynamic organelles that continuously undergo fusion and fission, we examined whether Piezo1 could regulate mitochondrial dynamics. Surprisingly, excessive mitochondrial fragmentation was observed after Yoda1 treatment, while GsMTx4 improved the morphology of mitochondria in CEP cells, as visualized by MitoTracker Red staining (Figure 2i). Overall, our results suggested that Piezo1 induced mitochondrial dysfunction in CEP cells by activating mitochondrial fission.

### 3.4 CaMKII mediated the effects of Piezo1 on mitochondrial fragmentation and dysfunction

**FIGURE 3:**
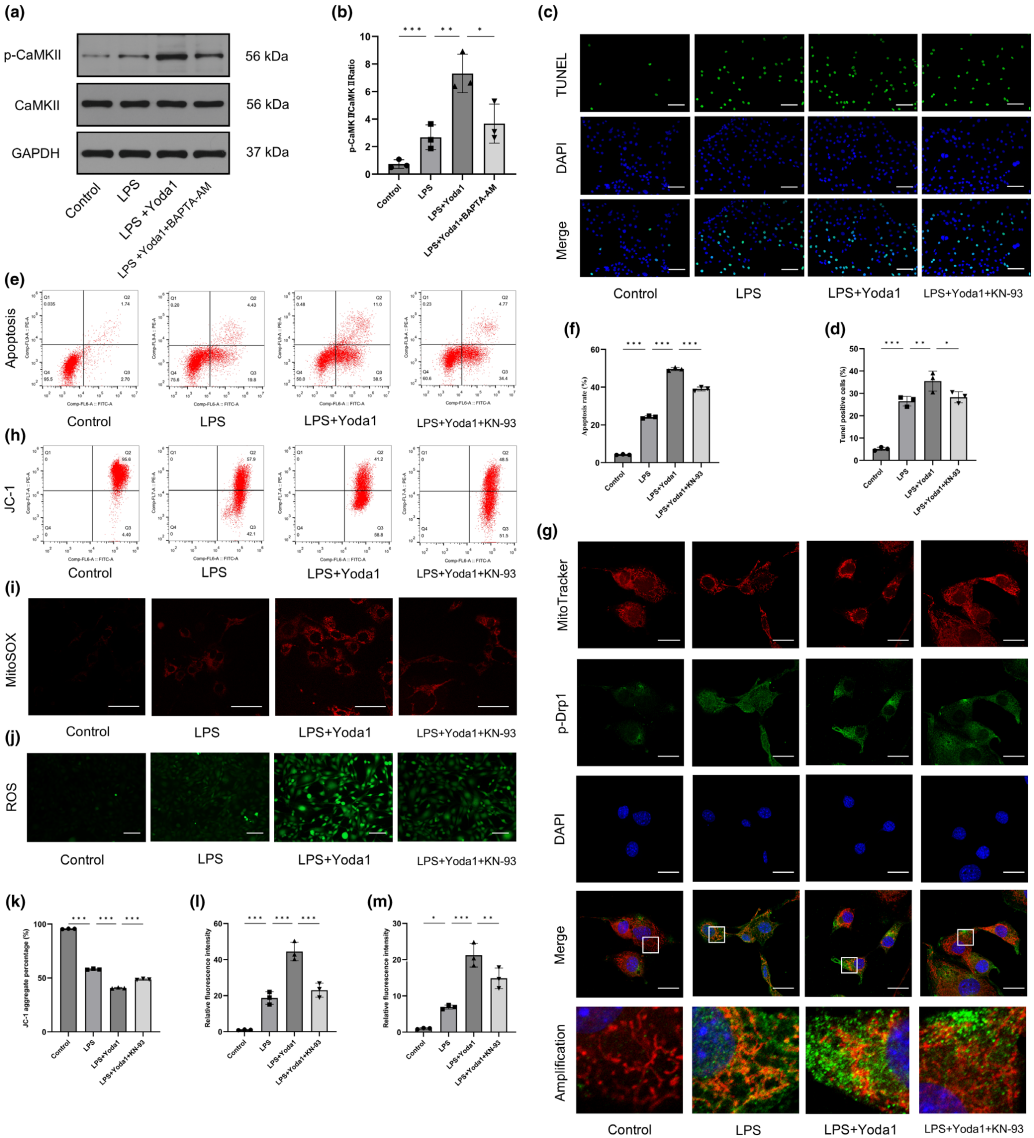
Piezo1 modulated mitochondrial morphology and function by influencing CaMKII activity. (a, b) western blot analysis showed that the level of CaMKII phosphorylation was regulated by Yoda1 and BAPTA‐AM. (c, d) TUNEL staining showed that KN‐93 partially reversed Yoda1‐induced cartilaginous endplate (CEP) cell apoptosis. Scale bar, 100 μm. (e, f) Flow cytometry with Annexin V‐FITC/PI verified the protective effects of KN‐93 on CEP cell apoptosis. (g) Double immunofluorescence staining indicated the colocalization of p‐Drp1 and MitoTracker Red. Scale bar, 25 μm. (h, k) Flow cytometry using JC‐1 to examine the MMP in CEP cells. (i, j, l, m) MitoSOX Red and DCFH‐DA staining were used to detect the production of mitochondrial and cellular reactive oxygen species (ROS) in CEP cells. Scale bars, 50 μm (I) and 100 μm (j). (*n* = 3 biological replicates, *p < 0.05; **p < 0.01; ***p < 0.001).

CaMKII, a serine/threonine kinase, plays an essential role in mediating the second messenger effects of Ca^2+^ in cells. However, most studies on CaMKII have focused on cardiovascular and neurologic diseases, and there is only a limited understanding of CaMKII in degenerative joint diseases (Reyes Gaido et al., 2023; Yasuda et al., 2022). We therefore examined the level of CaMKII phosphorylation, which is a measure of the CaMKII activation state, in CEP cells. Notably, we found that Yoda1 increased CaMKII phosphorylation at the Thr286 site, while knockdown of Piezo1 by siRNA significantly suppressed Yoda1‐induced CaMKII activation (Figure S1a,b). Accordingly, Yoda1 promoted Ca^2+^ influx in CEP cells, and the effect was reversed by Piezo1 silencing (Figure S1c,d). Under inflammatory conditions, CaMKII phosphorylation induced by Yoda1 was partially abolished by the Ca^2+^ chelator BAPTA‐AM (Figure 3a,b), suggesting that Ca^2+^/CaMKII signalling was regulated by Piezo1.

We next examined the effects of CaMKII on the phenotype of CEP cells by using the CaMKII inhibitor KN‐93. Flow cytometry and TUNEL staining showed that Yoda1 increased the number of apoptotic cells, while KN‐93 partially reversed the proapoptotic effects of Yoda1 (Figure 3c–f). Subsequently, we found that KN‐93 could protect mitochondrial function against the effects of Yoda1 in CEP cells under inflammatory conditions, which was confirmed by measuring MMP and mitochondrial and cellular ROS (Figure 3h–m).

Drp1 is known as a key mediator of mitochondrial fission. The activation of Drp1 depends on its phosphorylation and mitochondrial translocation. Thus, we hypothesized that CaMKII could facilitate Drp1 translocation from the cytosol to mitochondria and regulate mitochondrial dynamics in CEP cells. Double immunofluorescence staining verified that the colocalization of mitochondria and p‐Drp1 was enhanced after Yoda1 treatment (Figure 3g). However, this effect could be partially rescued by KN‐93. In summary, these data indicate that CaMKII is involved in the effects of Piezo1 on mitochondrial fission and dysfunction.

### 3.5 Piezo1 activated mitochondrial fission via the Ca^2+^/CaMKII/Drp1 axis

**FIGURE 4:**
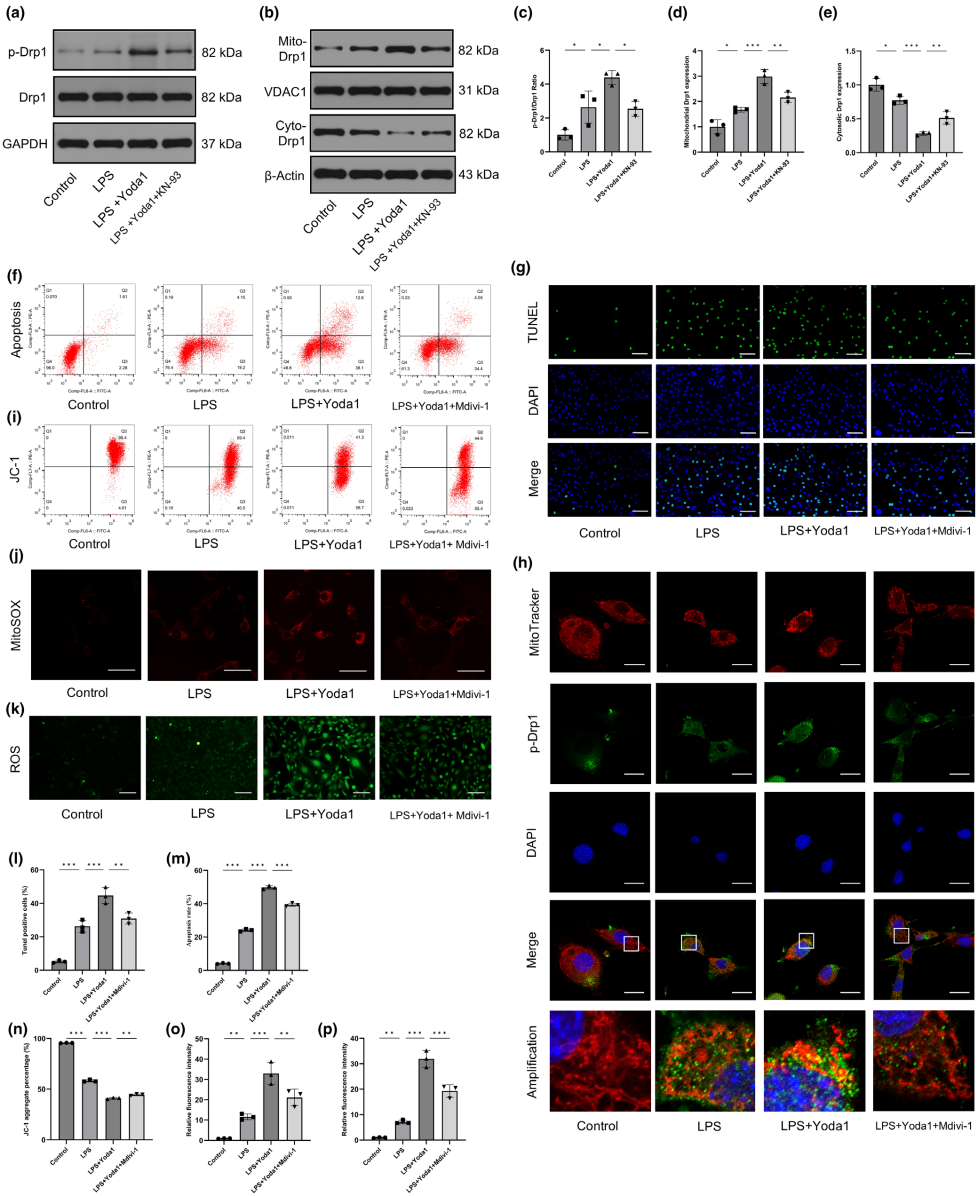
Piezo1 induced mitochondrial fission and dysfunction via the Ca^2+^/CaMKII/Drp1 axis in LPS‐treated cartilaginous endplate (CEP) cells. (a–e) The effects of KN‐93 on the phosphorylation and mitochondrial translocation of Drp1 were assessed using western blotting. (f, g, l, m) TUNEL staining and flow cytometry with Annexin V‐FITC/PI demonstrated that Mdivi‐1 partially rescued Yoda1‐induced CEP cell apoptosis. Scale bar, 100 μm. (h) The colocalization between p‐Drp1 and MitoTracker Red was verified by double immunofluorescence staining. Scale bar, 25 μm. (i, n) The MMP in CEP cells was evaluated by flow cytometry using JC‐1. (j, k, o, p) The production of mitochondrial and cellular reactive oxygen species (ROS) in CEP cells was measured by MitoSOX Red and DCFH‐DA staining, respectively. Scale bars, 50 μm (j) and 100 μm (k). (*n* = 3 biological replicates, *p < 0.05; **p < 0.01; ***p < 0.001).

Drp1 activity is modulated by posttranslational modifications. Phosphorylation of Drp1 at the S616 site enhances Drp1 activation and promotes its translocation from the cytosol to mitochondria. The western blot results indicated that Yoda1 increased the phosphorylation level of Drp1 at S616 (Figure 4a,c). Consistently, Drp1 was enriched in the mitochondrial fraction but depleted from the cytosolic fraction in response to Yoda1 in LPS‐treated CEP cells (Figure 4b,d,e). Additionally, KN‐93 partially prevented these changes. To understand the relationship between mitochondrial fission and Yoda1‐induced CEP cell phenotypes, the Drp1 inhibitor Mdivi‐1 was used (Cassidy‐Stone et al., 2008). The results revealed that Mdivi‐1 alleviated Yoda1‐induced apoptosis in CEP cells under inflammatory conditions (Figure 4f,g,l,m). Moreover, Mdivi‐1 partially counteracted Yoda1‐induced mitochondrial dysfunction in CEP cells, as indicated by the increase in MMP and reduced production of mitochondrial and cellular ROS (Figure 4i–k and n–p). To confirm the inhibitory effects of Mdivi‐1 on Drp1 activation and mitochondrial fission, double immunofluorescence staining was performed. The results demonstrated that Mdivi‐1 partially mitigated Yoda1‐induced Drp1 mitochondrial translocation and mitochondrial fission in CEP cells (Figure 4h). Together, these data indicated that Piezo1 aggravated inflammation‐induced CEP cell apoptosis by enhancing mitochondrial fission and dysfunction through the Ca^2+^/CaMKII/Drp1 axis.

### 3.6 Knockdown of Piezo1 abolished Drp1‐dependent mitochondrial fission

**FIGURE 5:**
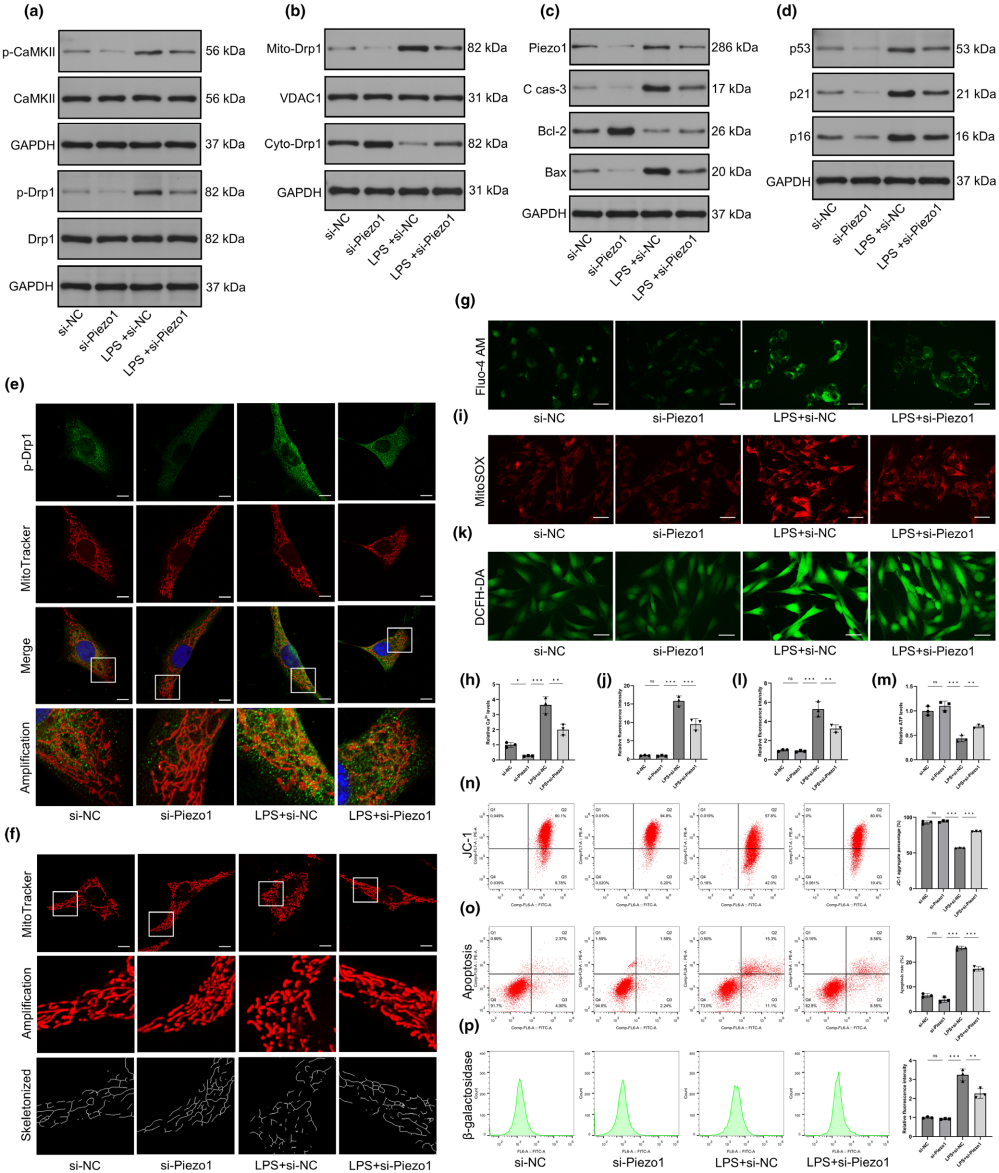
Knockdown of Piezo1 inhibited Drp1‐dependent mitochondrial fission. (a) The level of CaMKII phosphorylation and Drp1 phosphorylation after si‐Piezo1 treatment was measured by western blot analysis. (b) The subcellular localization of Drp1 was detected by western blotting. (c, d) The expression levels of apoptosis‐related and senescence‐related proteins were measured by western blot analysis. (e) The colocalization of Drp1 and mitochondria was shown by immunofluorescence staining and confocal microscopy. Scale bar, 10 μm. (f) The mitochondrial morphology was demonstrated by MitoTracker Red staining. Scale bar, 10 μm. (g, h) The intracellular Ca^2+^ levels were analysed by using the specific Ca^2+^‐sensitive fluorescent indicator Fluo‐4 AM. Scale bar, 40 μm. (i–l) The accumulation of mitochondrial and cellular reactive oxygen species (ROS) in cartilaginous endplate (CEP) cells was measured by MitoSOX Red and DCFH‐DA staining, respectively. Scale bars, 40 μm (i) and 40 μm (k). (m) Relative adenosine triphosphate (ATP) productions in CEP cells treated by si‐Piezo1. (n) The MMP was evaluated by flow cytometry using JC‐1. (o) Flow cytometry with Annexin V‐FITC/PI showed that si‐Piezo1 partially rescued LPS‐induced CEP cell apoptosis. (p) Cellular senescence was analysed by using flow cytometry with β‐galactosidase staining. (*n* = 3 biological replicates, *p < 0.05; **p < 0.01; ***p < 0.001).

Genetic knockdown of Piezo1 was achieved using the siRNA technique to validate the necessity of Piezo1 in modulating the translocation of Drp1 and mitochondrial phenotypes. Western blot analysis showed that Piezo1 silencing decreased the level of phosphorylated CaMKII, thereby suppressing Drp1 phosphorylation at S616 in LPS‐induced CEP cells (Figure 5a). Correspondingly, knockdown of Piezo1 promoted the redistribution of mitochondrial Drp1 to the cytosol (Figure 5b). The LPS‐induced upregulation of cleaved caspase‐3 and Bax and downregulation of Bcl‐2 were reversed by Piezo1 silencing (Figure 5c). The expression of senescence markers was also decreased by si‐Piezo1 (Figure 5d). The reduced colocalization of p‐Drp1 and mitochondria was validated by confocal microscopy (Figure 5e) MitoTracker staining demonstrated that si‐Piezo1 elongated mitochondrial branches (Figure 5f). Moreover, Yoda1 promoted mitochondrial fragmentation, but si‐Piezo1 could rebuild the mitochondrial networks (Figure S1e). Fluo‐4 AM staining indicated that Ca^2+^ influx was inhibited in CEP cells after Piezo1 knockdown (Figure 5g,h). Piezo1 silencing diminished mitochondrial and cellular ROS production under inflammatory conditions (Figure 5i–l). The siRNA to Piezo1 elevated ATP contents and rescued MMP collapse in LPS‐induced CEP cells (Figure 5m,n), thus si‐Piezo1 restored mitochondrial function. Increased apoptosis induced by Yoda1 was partially prevented by downregulating Piezo1 (Figure S1f,g and Figure 5o). β‐galactosidase staining showed that si‐Piezo1 alleviated cellular senescence in NPCs after LPS stimulation (Figure 5p). These results indicated that knockdown of Piezo1 could protect CEP cells under inflammatory conditions.

### 3.7 Inhibition of Piezo1 attenuated CEP degeneration in vivo

**FIGURE 6:**
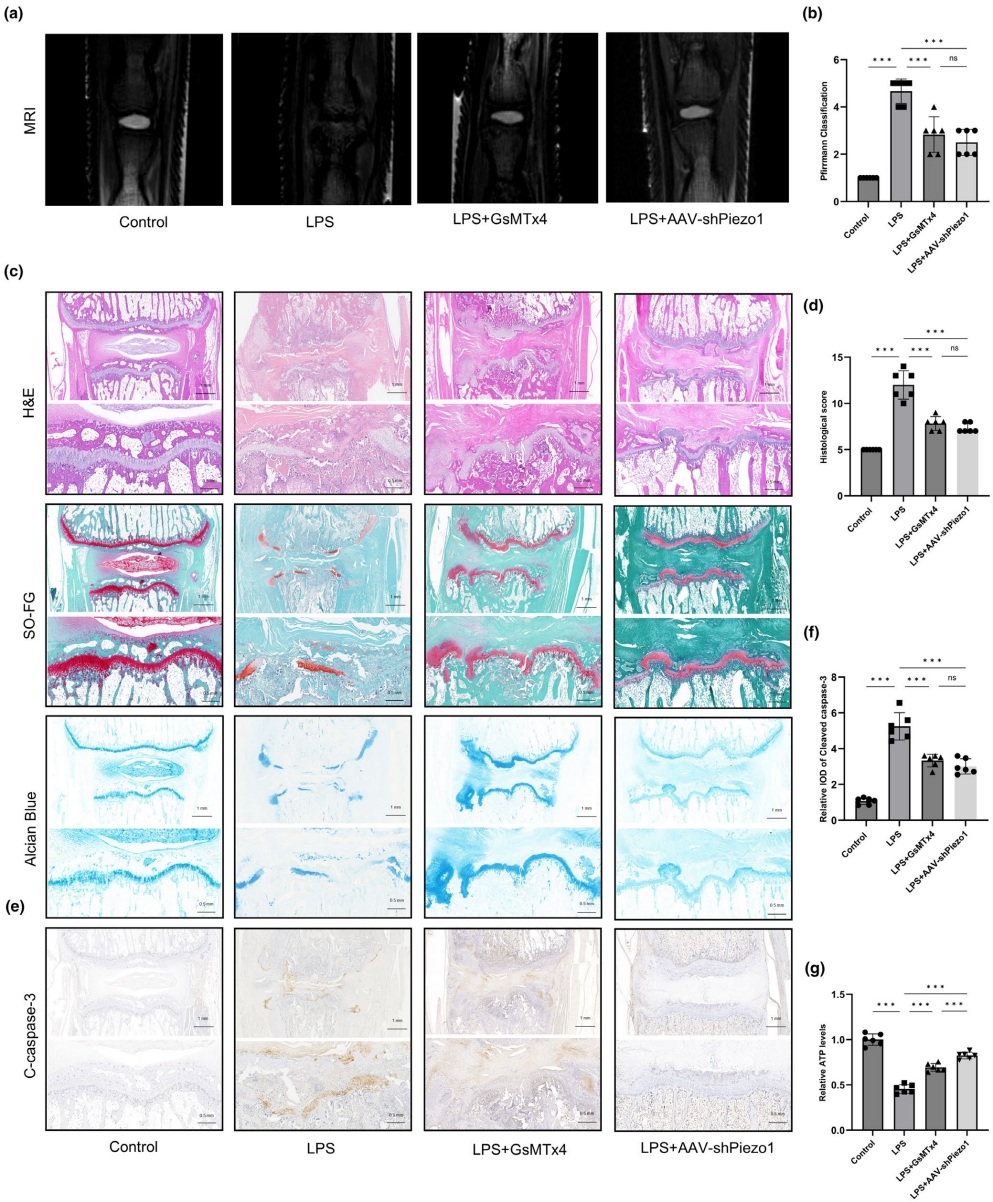
Inhibition of Piezo1 by GsMTx4 or AAV‐shPiezo1 attenuated inflammation‐induced cartilaginous endplate (CEP) degeneration in vivo. (a, b) The coccygeal vertebrae of rats were examined by magnetic resonance imaging (MRI). (c) H&E staining, Safranin O‐Fast Green staining and Alcian Blue staining of rat IVD samples. (d) Evaluation of IVDD by histological score. (e, f) Immunohistochemistry of cleaved caspase‐3. (g) Relative adenosine triphosphate (ATP) contents in IVD tissues. (*n* = 6 biological replicates, ***p < 0.001).

To mimic inflammation‐induced CEP degeneration, we established a rat model by subendplate injection of LPS. Genetic knockdown of Piezo1 was achieved by using AAV vectors containing short hairpin RNA (shRNA). The rats were divided into control, LPS, LPS + GsMTx4 and LPS + AAV‐shPiezo1 groups. After 4 weeks, the Pfirrmann grade of the IVDs was higher in the LPS group than in the control group (Figure 6a,b). Histological analyses were performed by H&E staining, Safranin O‐Fast Green staining and Alcian Blue staining. H&E staining showed that in rats in the control group, the NP, AF and CEP maintained normal morphology (Figure 6c,d). In the LPS group, the NP was degenerated, and the AF structure was disordered. Safranin O‐Fast Green and Alcian Blue staining further verified the alterations in the CEP. In the control group, the structure of the CEP was intact, and the arrangement of chondrocytes was tight. However, in the LPS group, significant CEP damage and loss of chondrocytes were observed. Surprisingly, GsMTx4 or AAV‐shPiezo1 treatment partially attenuated these signs of CEP degeneration and IVDD. Consistent with the in vitro results, the expression of cleaved caspase‐3 was elevated in the LPS group, while GsMTx4 or AAV‐shPiezo1 treatment reduced this expression (Figure 6e,f). Compared with the LPS group, inhibition of Piezo1 rescued ATP contents in IVD tissues (Figure 6g). Western blotting demonstrated that the expression of cytochrome c in the IVD tissues was upregulated by LPS stimulation, while GsMTx4 or AAV‐shPiezo1 treatment could decreased cytochrome c levels (Figure S2a). These results demonstrated that inhibition of Piezo1 could delay the progression of inflammation‐induced CEP degeneration in vivo.

## 4 DISCUSSION

IVDD is a complex and multifactorial process. Most studies have focused on the NP since herniation of the NP can lead to radiculopathy and is one of the most common indications for spine surgery worldwide. However, the CEP and AF are also involved in the pathological process of IVDD. In the present study, we focused on the CEP because it is a vital nutrient channel for the disc. Bibby et al. (2004) examined the effects of changes in glucose and oxygen concentrations and pH on the viability and metabolism of NP cells. The results indicated that nutrient deprivation limited NP cell survival. Yuan et al. (2015) established an IVDD model induced by subendplate injection of absolute ethanol. After injection, the microcirculation of the endplate was disrupted, causing ischaemia and a lack of nutrient supply, ultimately resulting in IVDD. CEP chondrocyte senescence and death impair the transport of nutrients in the disc. IVDD was accelerated due to the decline in nutrient supply. Clinically, Modic changes (MCs) are important signs of CEP degeneration and are closely associated with IVDD. MCs refer to signal intensity changes in the vertebral endplate and subchondral bone marrow, which can be identified by MRI (Modic et al., 1988). Three types of MCs have been described: MC type 1, which represents inflammation; MC type 2, which represents lipid marrow replacement; and MC type 3, which represents calcification (Udby et al., 2022). A growing body of evidence has suggested that MCs are secondary to inflammation and infection (Crockett et al., 2017; Rajasekaran et al., 2022). However, the pathogenetic mechanisms of MCs and CEP degeneration are largely unexplored. Therefore, we established in vitro and in vivo models of inflammation‐induced CEP degeneration with LPS. Our results confirmed that LPS induced CEP cell senescence and apoptosis. And subendplate injection of LPS led to CEP degeneration in rats.

Piezo1 is a newly discovered mechanosensitive cation channel that has increasingly attracted attention in the field of musculoskeletal diseases. Studies have shown that Piezo1 is stably expressed and has essential functions in mesenchymal stem cells, osteoblasts, osteocytes, chondrocytes and NP cells (Qin et al., 2021). Recently, Wang et al. (2021) reported that Piezo1 was upregulated by increased ECM stiffness and contributed to oxidative stress and endoplasmic reticulum stress in NP cells. Inhibiting Piezo1 alleviated senescence and apoptosis in NP cells under high ECM stiffness. Similarly, Wu et al. (2022) revealed that Piezo1 activated NF‐kB p65 and accelerated ECM stiffness‐induced senescence in NP cells. These researchers set mechanical factors as a starting point to study the functions of Piezo1 in IVDD. However, inspired by the elegant work of Lee et al. (2021), we used LPS‐induced inflammation as a starting point. We hypothesized that LPS increases the basal expression level of Piezo1 and then facilitates Piezo1‐mediated downstream intracellular signal transduction. In the present study, we found that Piezo1 protein levels were increased in degenerated CEP tissues and that LPS upregulated Piezo1 expression transcriptionally and translationally in CEP cells, which is consistent with the results observed by Velasco‐Estevez et al. (2020) and Liu et al. (2023) in nonmusculoskeletal diseases. Both activation and inhibition of Piezo1 contributed to the regulation of CEP cell senescence and apoptosis under inflammatory conditions. Subendplate injection of GsMTx4 or AAV‐shPiezo1 attenuated LPS‐induced CEP degeneration in a rat model. Taken together, these data demonstrate that Piezo1 is a mediator of inflammation‐induced CEP degeneration. Although we only used Yoda1 to mimic Piezo1 activation by mechanical stimuli in the experiments, it is well known that the CEP is constantly exposed to mechanical forces, and abnormal stress causes CEP degeneration (Crockett et al., 2017). Indeed, more evidence is needed to further determine how inflammatory signalling upregulates Piezo1 expression and whether it leads to mechanical hypersensitivity of CEP cells.

Maintaining mitochondrial homeostasis and preventing mitochondrial dysfunction have been recognized as a crucial strategy to delay the progression of IVDD, but the role of mitochondrial fission in IVDD are less discussed (Song et al., 2021). Our previous study showed that mitochondrial fission, which is induced by inflammation, mechanical loading or oxidative stress, participates in apoptosis, inflammatory responses and ECM metabolism in IVDD (Lin et al., 2023). Dynamin‐related protein 1 (Drp1), a member of the dynamin family of guanosine triphosphate (GTP)‐binding proteins, is the key mediator of mitochondrial fission. Primarily residing in the cytosol, Drp1 is activated and recruited to the OMM of mitochondria when fission is initiated by pathological stimuli. After recruitment, Drp1 oligomerizes and forms a ring‐like structure to cleave mitochondria. The activation of Drp1 is modulated by posttranslational modifications. Phosphorylation of Drp1 at serine 616 enhances Drp1 activity and promotes its translocation from the cytosol to mitochondria (Jin et al., 2021). An increasing number of studies have shed light on the relationship between mitochondrial dynamics and IVDD (Lin et al., 2023). Hu et al. (2022) found that compression induced excessive mitochondrial fragmentation and dysfunction, consequently leading to NP cell apoptosis. Compression upregulated the expression of Drp1 and other mitochondrial fission‐related proteins while suppressing fusion‐related proteins. Heat shock protein 70 (HSP70), a cytoprotective protein, improved mitochondrial morphology and function by promoting Sirtuin‐3 (SIRT3) expression. Peng et al. (2022) used LPS to mimic the inflammatory microenvironment of IVDD. LPS triggered NP cell apoptosis and pyroptosis by impairing mitochondrial dynamics and mitophagy. Increased mitochondrial translocation of Drp1 was observed in LPS‐treated NP cells. A20/TNFAIP3, a zinc finger protein, inhibited LPS‐induced excessive mitochondrial fission and cell death. However, in these studies, posttranslational modifications of Drp1 were not investigated. The present study revealed that the mitochondrial translocation of Drp1 was promoted by Drp1 phosphorylation at the S616 site in CEP cells under inflammatory conditions. Surprisingly, Piezo1 enhanced CaMKII phosphorylation by triggering Ca^2+^ influx, and the phosphorylation level of Drp1 was elevated by activated CaMKII in CEP cells, which is consistent with previous findings (Xu et al., 2016). Collectively, our results indicate a downstream pathway of Piezo1 that links Ca^2+^/CaMKII and Drp1 to bridge Ca^2+^ signalling with mitochondrial fission in CEP degeneration.

In summary, our work identified that Piezo1 was upregulated and involved in the regulation of CEP cell senescence and apoptosis under inflammatory conditions. Mechanistically, Piezo1 aggravated mitochondrial fragmentation and dysfunction by activating the Ca^2+^/CaMKII/Drp1 axis. Inhibition or knockdown of Piezo1 attenuated inflammation‐induced CEP degeneration in vivo. Targeting Piezo1 may be a promising therapeutic strategy against CEP degeneration and IVDD in the future.

## AUTHOR CONTRIBUTIONS

Z.L., J.J. and F.Z. were responsible for the study design. Z.L., G.X. and X.L. conducted the study and collected data. Z.L., G.X., X.L., H.W., F.L., X.X. and J.S. analysed and interpretated the data. All authors were involved in the original draft preparation. J.S., J.J., X.M. and F.Z. reviewed and edited the manuscript. J.J., X.M. and F.Z. supervised the project. All authors have read and approved the manuscript.

## CONFLICT OF INTEREST STATEMENT

The authors declare that they have no conflict of interest.

## Supporting information


Figure S1.
**Figure S2**.

Table S1.

## Data Availability

The data that support the findings of this study are available in the main text or the Supplementary Materials or from the corresponding author upon reasonable request.
