# Supplementary material for: Piezo1 exacerbates inflammation‐induced cartilaginous endplate degeneration by activating mitochondrial fission via the Ca2+/CaMKII/Drp1 axis
Source: Aging Cell. 2024 Nov 28;24(4):e14440. doi: 10.1111/acel.14440 (PMC11984661; doi:10.1111/acel.14440)
Supplement: Supplementary file 1 — Figure S1. Figure S2. [file ACEL-24-e14440-s002.docx]

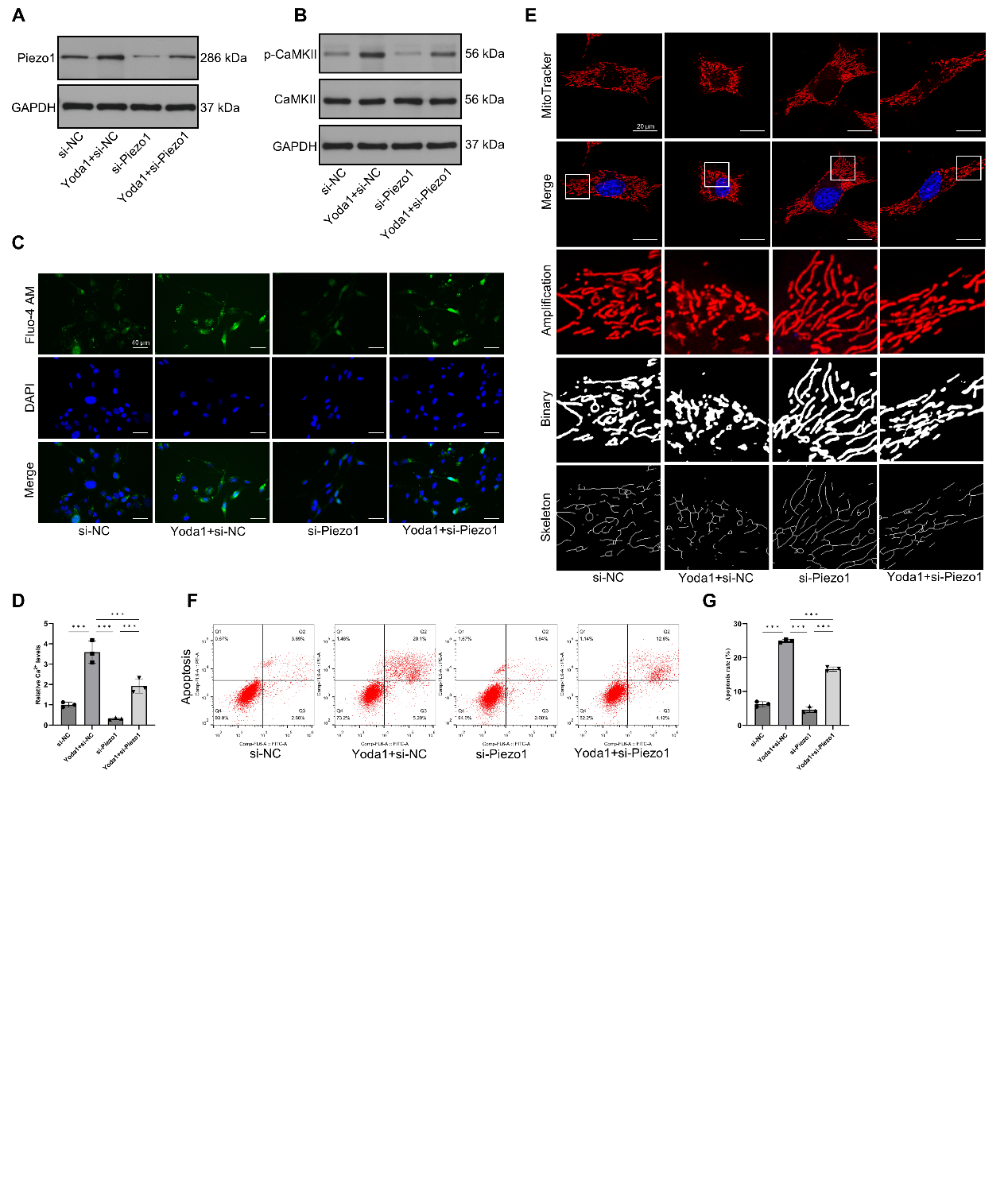


**Fig. S1** Knockdown of Piezo1 abolished Piezo1-induced CaMKII phosphorylation and mitochondrial fragmentation. (A, B) Western blot analysis of the expression levels of Piezo1 and p-CaMKII/CaMKII in CEP cells after treated with Yoda1 and si-Piezo1. (C, D) Intracellular Ca^2+^ levels were detected using the specific Ca^2+^-sensitive fluorescent indicator Fluo-4 AM. Scale bar, 40 μm. (E) The mitochondrial morphology in CEP cells was analysed by MitoTracker Red staining. Scale bar, 20 μm. (F, G) CEP cell apoptosis was measured by flow cytometry with Annexin V-FITC/PI. (n = 3 biological replicates, *p < 0.05; **p < 0.01; ***p < 0.001)


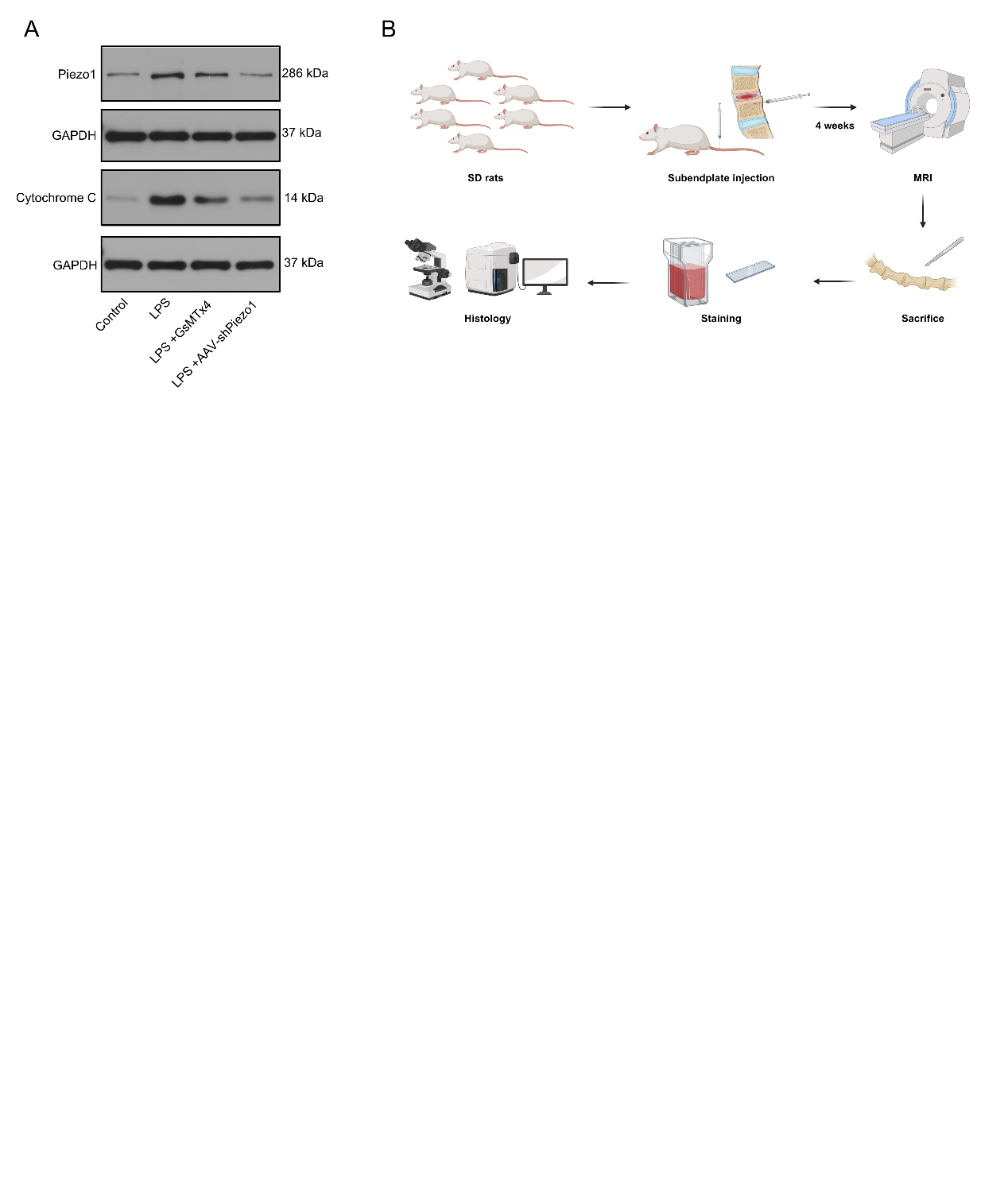


**Fig. S2** (A) The expression of Piezo1 and cytochrome c in the IVD tissues were assessed by western blotting. (B) The flow chart of the experimental design. (n = 6 biological replicates, *p < 0.05; **p < 0.01; ***p < 0.001)
